# Phage–Antibiotic–Peptide Synergy Overcomes Biofilm-Mediated Multidrug Resistance in *Serratia marcescens*

**DOI:** 10.3390/antibiotics15090879

**Published:** 2026-09-08

**Authors:** Aryaan P. Duggal, Adit B. Alreja, Isha Vashee, Hayley Nordstrom, Erin Harrelson, Nakia Fallen, Kari-Ann Takano, Ryan A. Blaustein, Derrick E. Fouts, Norberto Gonzalez-Juarbe

**Affiliations:** 1 Department of Cell Biology and Molecular Genetics, University of Maryland, College Park, MD 20742, USA; 2 Human Genomic Medicine, J. Craig Venter Institute, Rockville, MD 20854, USA; 3 Department of Nutrition and Food Science, University of Maryland, College Park, MD 20742, USA

**Keywords:** phage, antibiotic, antimicrobial peptides, biofilms, *Serratia marcescens*

## Abstract

Background/Objectives*: Serratia marcescens* is an opportunistic pathogen that causes severe hospital-acquired infections, notable for its biofilm formation abilities and development of extensive antibiotic resistance. Here, we aim to evaluate the efficacy of bacteriophages, antibiotics, and antimicrobial peptides (BAP), alone and in combination, against fourteen multidrug-resistant (MDR) *S. marcescens* isolates sourced from hospitals and other environmental settings. Methods: S. marcescens was grown planktonically or in surface-associated biofilms, and biofilm biomass was measured via changes in absorbance and colony-forming units or live/death staining. Results: Combining bacteriophage with a low-dose cocktail of penicillin–streptomycin, kanamycin, and ciprofloxacin enhanced antimicrobial activity compared with antibiotics alone. Across the isolate panel, responses to BAP treatment varied according to determined antibiotic resistance profiles. The highly resistant AR-0517 isolate was selected for detailed mature biofilm analysis, where the BAP treatment reduced biofilm biomass by 97.8% and recoverable bacteria by 99.99%. Microscopy and viability assays further confirmed extensive biofilm disruption and bacterial killing. Conclusions: These findings demonstrate that simultaneous targeting of multiple bacterial pathways can enhance antimicrobial activity against MDR S. marcescens in vitro and support further evaluation of BAP as a potential strategy for biofilm-associated infections.

## 1. Introduction

*Serratia marcescens* is a Gram-negative, facultative anaerobic bacterium within the Enterobacterales order. Historically considered an environmental organism, with reservoirs spanning soil, plants, and surface water, it has become a widespread opportunistic pathogen responsible for numerous hospital-associated infections, particularly in immunocompromised patients, the elderly, and neonates [1]. The ability of *S. marcescens* to establish biofilms and persist in hospital environments, such as on medical equipment, has led to frequent outbreaks in intensive care units (ICUs) and neonatal intensive care units (NICUs), which have proven to be deadly to patients [2,3]. Epidemiological studies have documented the increasing burden of *S. marcescens* infections in healthcare settings [4,5]. Notably, neonatal and geriatric patients are disproportionately affected by nosocomial infections, including sepsis caused by *S. marcescens*. These patients suffer from higher mortality rates, longer-term morbidity, and increased costs of care [6]. These resulting complications are significantly worsened in infections caused by strains of *S. marcescens* that exhibit resistance to a broad spectrum of antibiotics [7,8].

*S. marcescens* exhibits both intrinsic and acquired antibiotic resistance mechanisms, complicating treatment strategies. Intrinsically, the bacterium possesses efflux pumps and reduced outer membrane permeability, limiting antibiotic uptake [7]. Acquired resistance arises through horizontal gene transfer and mutations affecting antibiotic targets. For example, mutations in DNA gyrase (*gyrA*, *gyrB*) can confer resistance to fluoroquinolones, while ribosomal modifications reduce aminoglycoside efficacy [9,10]. A study conducted by Bryant et al. showed that *S. marcescens* gained a bla_KPC-4_ gene from *Enterobacter cloacae* via a plasmid through a wound in a hospital-acquired infection of a patient, highlighting the acquisition of antimicrobial resistance genes [11]. In addition, some strains of *S. marcescens* can produce β-lactamases that degrade β-lactam antibiotics, including penicillins and cephalosporins [12]. *S. marcescens* environmental persistence and acquisition of antimicrobial resistance genes are enhanced by a defining virulence trait: its ability to form biofilms, which are structured bacterial communities surrounded by an extracellular matrix composed of polysaccharides, proteins, and extracellular DNA [13]. Biofilm formation facilitates colonization of inanimate surfaces, particularly moist areas such as sinks and drains, but also medical devices such as central venous catheters, endotracheal tubes, and ventilators [14,15]. Within biofilms, bacteria exhibit an increased resistance to host immune responses and antimicrobial agents [16,17]. Studies indicate that biofilm-associated *S. marcescens* infections require antibiotic concentrations significantly higher than planktonic infections, often exceeding clinically feasible doses for safe use in patients [8,18]. The resilience of *S. marcescens* biofilms, and that of other Enterobacterales members, poses significant challenges for hospital sanitation protocols, such as standard disinfectants including bleach, ammonium chloride, and other chlorine-based cleaning agents [15]. Moreover, biofilms can persist on surfaces for extended periods, even under stringent cleaning regimens, contributing to recurrent outbreaks in healthcare settings [4,14,19,20,21,22]. Taken together, these findings indicate that it is imperative to design new strategies to decolonize biofilms and mitigate the risk of S. marcescens spread in hospital settings.

Despite adherence to standard sanitation protocols, current disinfection strategies can be inadequate for the eradication of *Serratia* spp. biofilms in healthcare environments, necessitating the development of novel approaches. Widely used agents such as diluted bleach exhibit limited efficacy against mature biofilms due to poor penetration of the extracellular matrix and rapid neutralization in organic-rich environments [23]. Similarly, quaternary ammonium compounds (QACs) have been shown to reduce planktonic bacterial loads while leaving biofilm-embedded organisms largely viable [24]. Biocide and antibiotic resistance genes frequently occur together, and biocide chemical residues on environmental surfaces may select for “qac” antimicrobial resistance genes [25]. Hydrogen peroxide vapor systems, though more effective in some contexts, require prolonged exposure and environmental containment to exert bactericidal effects, which limits their practical utility in high-turnover clinical settings [26]. In situ investigations have confirmed the persistence of biofilm-forming *S. marcescens* strains on surfaces that were considered disinfected, contributing to repeated outbreaks in NICUs and intensive care units [27]. Antibiotic lock therapy in combination with systemic antibiotic therapy has recently been found to be an effective way to treat peripherally inserted central venous catheter-related bloodstream infections [28]. However, the complexity of the biofilm’s organic architecture, combined with the bacterium’s innate and acquired resistance mechanisms, renders most standard sanitation efforts insufficient. These limitations underscore the need for engineered combination therapies that are multifaceted in their mechanism of eradication and employ a multidrug approach to effectively eliminate *S. marcescens*.

In pursuit of such multifaceted therapies, we set out to screen the effectiveness of major antibiotics against multidrug-resistant (MDR) *S. marcescens* both in planktonic and biofilm forms. We found that different classes of antibiotics exhibited varying levels of killing activity on planktonic and biofilm-associated *S. marcescens*. A combination regimen using a low-dose antibiotic cocktail showed dose-dependent efficacy in reducing planktonic bacterial growth and biofilm biomass. As natural predators of bacteria, phages have evolved to selectively kill target bacteria by hijacking their biological machinery. A growing body of evidence suggests that in some situations, phages can be utilized synergistically with antibiotics to improve antimicrobial effectiveness. Our results showed that a combination of bacteriophage and antibiotics reduced biofilm biomass to a greater degree than the antibiotic mixture alone, highlighting the synergistic effect of phage therapy and antibiotic treatment. To take our findings further, we employed a Bacteriophage + Antibiotics + Antimicrobial Peptides (BAP) approach and found that this triple treatment was the most effective at decolonizing biofilms, especially on MDR *S. marcescens*. Taken together, our findings highlight the importance of multidimensional antimicrobial approaches to target *S. marcescens*-associated infections.

## 2. Results

### 2.1. Distinct Antibiotic Classes Exert Variable Inhibitory Effects on S. marcescens in Planktonic and Biofilm Forms

**Figure 1:**
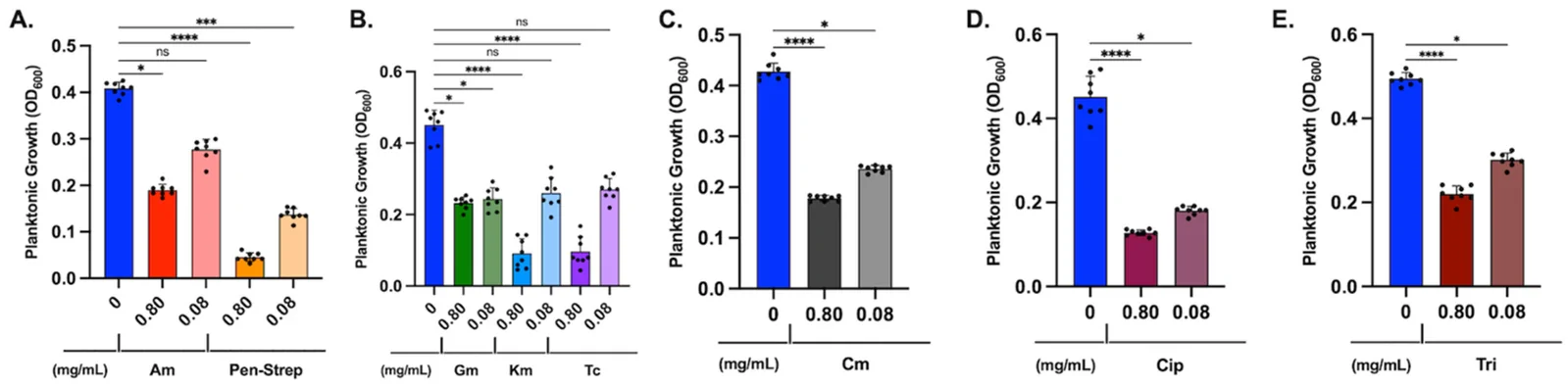
Major antibiotic classes have differential efficacy against planktonic *S. marcescens*. *S. marcescens* UT-383 was grown planktonically to mid-log phase and treated for 24 h with antibiotics at 0.80 or 0.08 mg/mL. (**A**) ampicillin and penicillin–streptomycin; (**B**) gentamicin, kanamycin, and tetracycline; (**C**) chloramphenicol; (**D**) ciprofloxacin; and (**E**) trimethoprim. Planktonic growth was quantified by OD600. Statistical differences were determined using the Kruskal–Wallis test followed by Dunn’s multiple-comparison post-test. * *p* ≤ 0.05, *** *p* ≤ 0.001, **** *p* ≤ 0.0001, ns = no significance. Experiments were performed using three independent biological replicates with eight technical replicates per biological replicate. Error bars denote standard deviation.

To compare the relative inhibitory activity of different antibiotic classes, we performed an initial high- and low-dose antimicrobial screen using 0.80 and 0.08 mg/mL, respectively, against *S. marcescens* UT-383. These concentrations were selected based on our previous *S. marcescens* biofilm study [8], in which chloramphenicol was evaluated at elevated concentrations, including 0.08 and 0.80 mg/mL, against established biofilms. In that study, 0.08 mg/mL reduced preformed biofilm biomass, whereas 0.80 mg/mL additionally produced a marked reduction in bacterial viability. We therefore carried these two concentrations forward as standardized low- and high-concentration screening conditions to enable comparative evaluation across antibiotic classes in the present study. These concentrations were used as comparative biofilm-screening exposures and were not intended to represent conventional antimicrobial susceptibility testing, MIC values, or clinically achievable systemic concentrations. Antibiotics targeting cell wall synthesis, protein synthesis, DNA replication, and folate synthesis were included in this comparative screen (Table S1). Treatment with β-lactam- and aminoglycoside-containing drugs, penicillin–streptomycin and ampicillin, at concentrations of 0.80 mg/mL and 0.08 mg/mL resulted in a substantial reduction in bacterial density, with penicillin–streptomycin exhibiting a lower OD_600_ at both concentrations and ampicillin exhibiting a less pronounced effect (Figure 1A). For aminoglycosides and tetracyclines, kanamycin and tetracycline at 0.80 mg/mL caused a large reduction in planktonic growth, while at 0.08 mg/mL the reduction was not significant. Gentamicin partially reduced planktonic growth at both doses. (Figure 1B). As previously observed by our group [8], chloramphenicol (an amphenicol) displayed dose-dependent inhibition of *S. marcescens* (Figure 1C). The fluoroquinolone ciprofloxacin and the antifolate trimethoprim also produced a dose-dependent decrease in optical density, with the higher dose providing over 50% reduction (Figure 1D,E). These findings reveal a clear pattern of variable susceptibility among planktonic S. *marcescens* to different antibiotic classes, underscoring the importance of employing combination strategies against bacterial targets.

**Figure 2:**
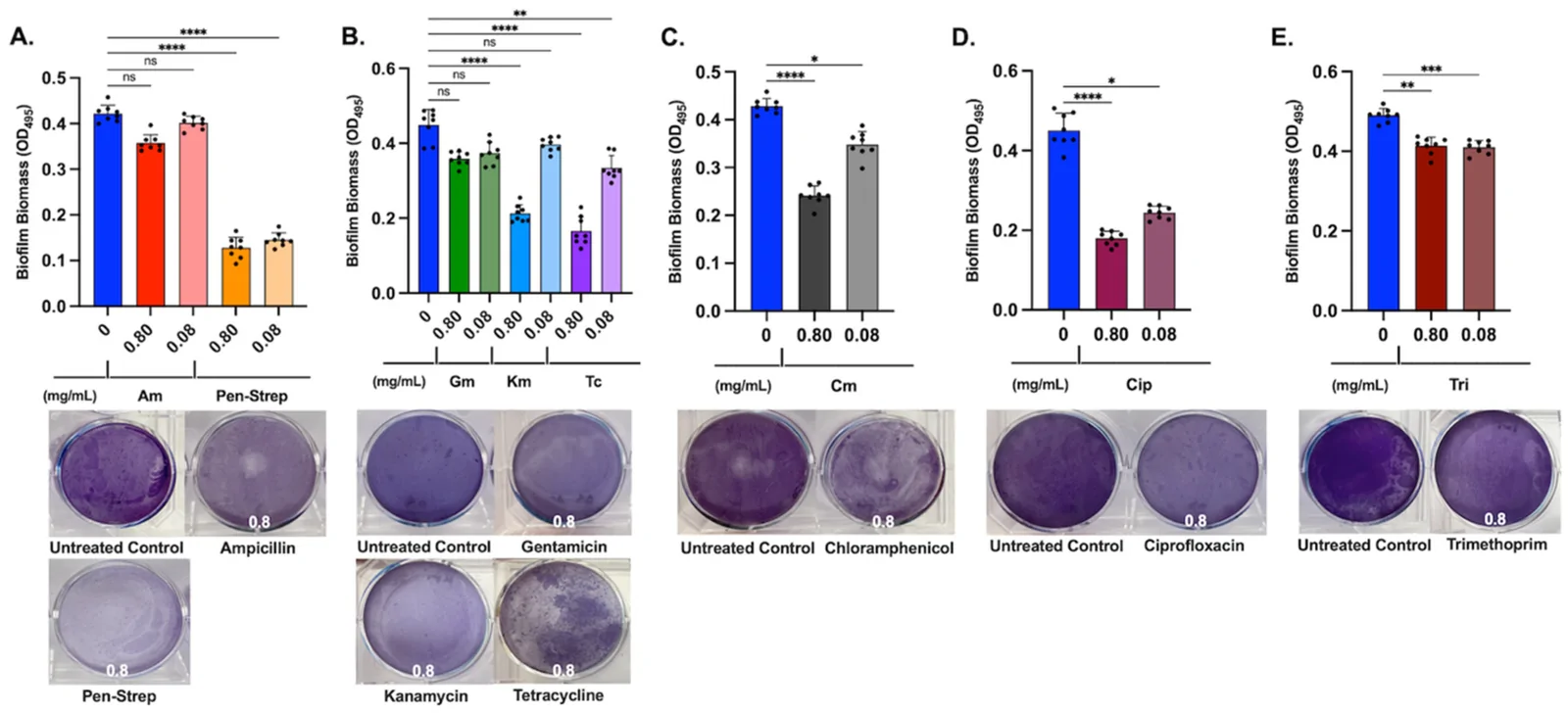
Major antibiotic classes have differential efficacy against mature *S. marcescens* biofilms. *S. marcescens* UT-383 biofilms were established for 48 h and subsequently treated for 24 h with antibiotics at 0.80 or 0.08 mg/mL. (**A**) ampicillin and penicillin–streptomycin; (**B**) gentamicin, kanamycin, and tetracycline; (**C**) chloramphenicol; (**D**) ciprofloxacin; and (**E**) trimethoprim. Biofilm biomass was quantified by crystal violet staining and absorbance at OD_490_. Statistical differences were determined using the Kruskal–Wallis test followed by Dunn’s multiple-comparison post-test. * *p* ≤ 0.05, ** *p* ≤ 0.01, *** *p* ≤ 0.001, **** *p* ≤ 0.0001, ns = no significance. Experiments were performed using three independent biological replicates with eight technical replicates per biological replicate. Error bars denote standard deviation.

Biofilms are known for providing bacteria with formidable resilience against antimicrobial agents and are relevant to device-associated infections, posing a greater therapeutic challenge [14,15,17]. Given this knowledge, we aimed to evaluate the activity of the different classes of antibiotics against highly structured mature biofilms. Using the high (0.80 mg/mL) and low (0.08 mg/mL) antibiotic doses shown in Figure 1 on *S. marcescens* UT-383, we observed that penicillin–streptomycin and ciprofloxacin (Figure 2A,D) were the most effective at each dose in reducing biofilm biomass. Ampicillin, chloramphenicol, kanamycin, and tetracycline (Figure 2A–C) showed a dose-dependent effect, and gentamicin along with trimethoprim only partially reduced biofilm biomass (Figure 2B,E).

### 2.2. Low-Dose Antibiotic Combinations Reduce Planktonic Growth and Biofilm Biomass of S. marcescens

**Figure 3:**
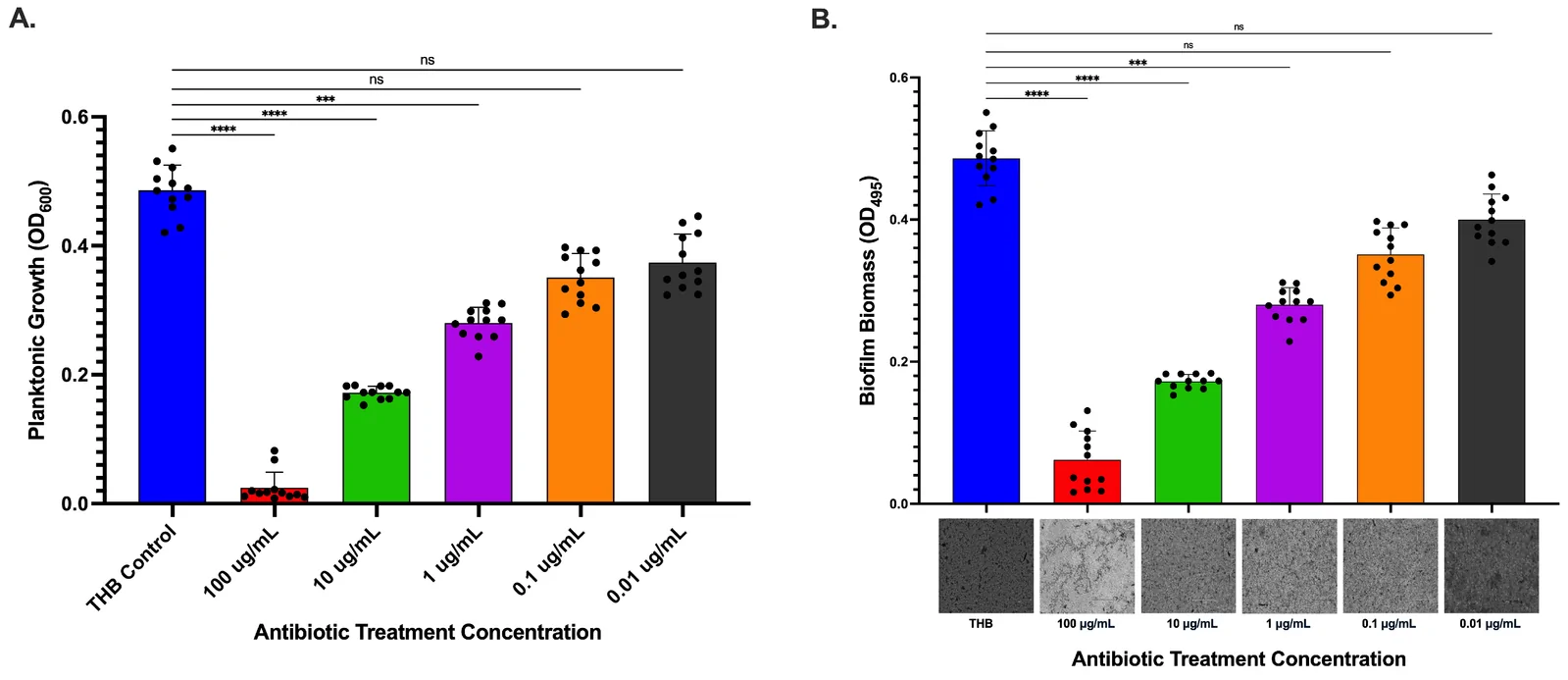
Antibiotic cocktail reduces planktonic growth and biofilm biomass in a dose-dependent manner. (**A**). Planktonic-grown bacteria were challenged mid-log with a combination mixture composed of three antibiotics (PenStrep, ciprofloxacin, and kanamycin) at concentrations between 100 µg/mL and 0.01 µg/mL, and absorbance at OD600 was measured after 24 h. (**B**). Biofilm biomass measured from crystal violet staining of mature biofilms treated with the antibiotic at concentrations between 100 µg/mL and 0.01 µg/mL for 24 h prior to staining and OD 495 absorbance reading. Statistical differences were determined by the Kruskal–Wallis test with Dunn’s multiple-comparison post-test. *** *p* ≤ 0.001, **** *p* ≤ 0.0001 (*n* = 12), ns = no significance. Error bars denote standard deviation.

Given the variation in antibiotic susceptibility observed, we sought to evaluate a low-dose antibiotic combination regimen rather than relying on a single high-dose antibiotic treatment. Since antibiotics also destroy commensal bacteria, a multidrug, lower-dose approach could potentially lessen these harmful effects on commensals. We combined penicillin–streptomycin, ciprofloxacin, and kanamycin at reduced concentrations to target multiple bacterial pathways. Penicillin–streptomycin disrupts cell wall and protein synthesis, ciprofloxacin inhibits DNA gyrase and topoisomerase IV, and kanamycin halts translation via inhibition of the 30S ribosomal subunit [29]. This multi-pronged strategy was evaluated by challenging logarithmic-phase planktonic bacteria with serial dilutions of an antibiotic cocktail composed of one-third penicillin–streptomycin, one-third ciprofloxacin, and one-third kanamycin by total antibiotic concentration. Because the penicillin–streptomycin component consisted of equal concentrations of penicillin G and streptomycin, the 100 µg/mL cocktail contained 16.65 µg/mL penicillin G, 16.65 µg/mL streptomycin, 33.3 µg/mL kanamycin, and 33.3 µg/mL ciprofloxacin. The highest dose of 100 µg/mL antibiotic cocktail reduced planktonic growth to near-background optical density levels, while ten-fold dilutions to 10 µg/mL, 1 µg/mL, 0.1 µg/mL, and 0.01 µg/mL showed a dose-dependent reduction in efficacy, with only 10 µg/mL and 1 µg/mL significantly reducing planktonic bacterial growth (Figure 3A).

To test the effectiveness of our antibiotic mixture against *S. marcescens* biofilms, we generated static biofilms and challenged them with five different doses of the antibiotic mixture ranging from 0.01 to 100 µg/mL, as was done for planktonic bacteria. Here we observed that the 100 and 10 µg/mL doses of our antibiotic mixture resulted in a major reduction in biofilm biomass. The 1 µg/mL, 0.1 µg/mL, and 0.01 µg/mL doses showed a dose-dependent reduction in effectiveness against *S. marcescens* biofilm. Microscopy images mirrored these results, showing disrupted biofilm architecture at higher concentrations and intact structures at lower doses (Figure 3B). Taken together, these data demonstrate that the antibiotic cocktail reduces planktonic growth and biofilm biomass of *S. marcescens* in a dose-dependent manner.

### 2.3. Isolation of S. marcescens Bacteriophages

While reduced-dose antibiotic strategies have been explored to limit antimicrobial exposure, their efficacy may remain constrained by incomplete bacterial clearance and the potential for adaptive resistance. Consequently, alternative antimicrobial approaches are needed, with bacteriophage therapy emerging as a promising strategy. We identified two bacteriophages (ϕJCVI_Sm1 and ϕJCVI_Sm2) from municipal wastewater that produced plaques on our laboratory MDR *S. marcescens* strain UT-383 and a non-MDR strain HER1170, grown planktonically on agar plates (Supplemental Figure S1A,B). The complete genomes of both phages were determined from assembly of Illumina reads, yielding single circular contigs of 74,559 bp and 183,012 bp for ϕJCVI_Sm1 and ϕJCVI_Sm2, respectively. ϕJCVI_Sm1 has the highest nucleotide similarity (i.e., BLASTN Max score) to *Gibbsiella* phage vb_Gq_GIB1 (Max score: 33,937, Query coverage: 80%, Percent identity: 93.04%, E-value: 0.0) and *Klebsiella* phage vB_KpnP_P184 (Max score: 32,817, Query coverage: 85%, Percent identity: 86.89%, E-value: 0.0), with the best match using Phage_Finder to model phage N4. A comparison of synteny and protein sequence identity showed relatedness of ϕJCVI_Sm1 to model *Escherichia* phage N4 (Supplemental Figure S1C). ϕJCVI_Sm2 has the highest nucleotide similarity to *Serratia* and *Cronobacter* phages, specifically to *Serratia* phage Incoe (Max score: 5304, Query coverage: 35%, Percent identity: 78.74%, E-value: 0.0) and *Cronobacter* phage LPCS28 (Max score: 4937, Query coverage: 27%, Percent identity: 76.68%, E-value: 0.0), while the best-matching *E. coli* model phage was T4 by Phage_Finder (Supplemental Figure S1D).

### 2.4. Combination of S. marcescens Bacteriophage and Antibiotic Cocktail Leads to Enhanced Biofilm Disruption

**Figure 4:**
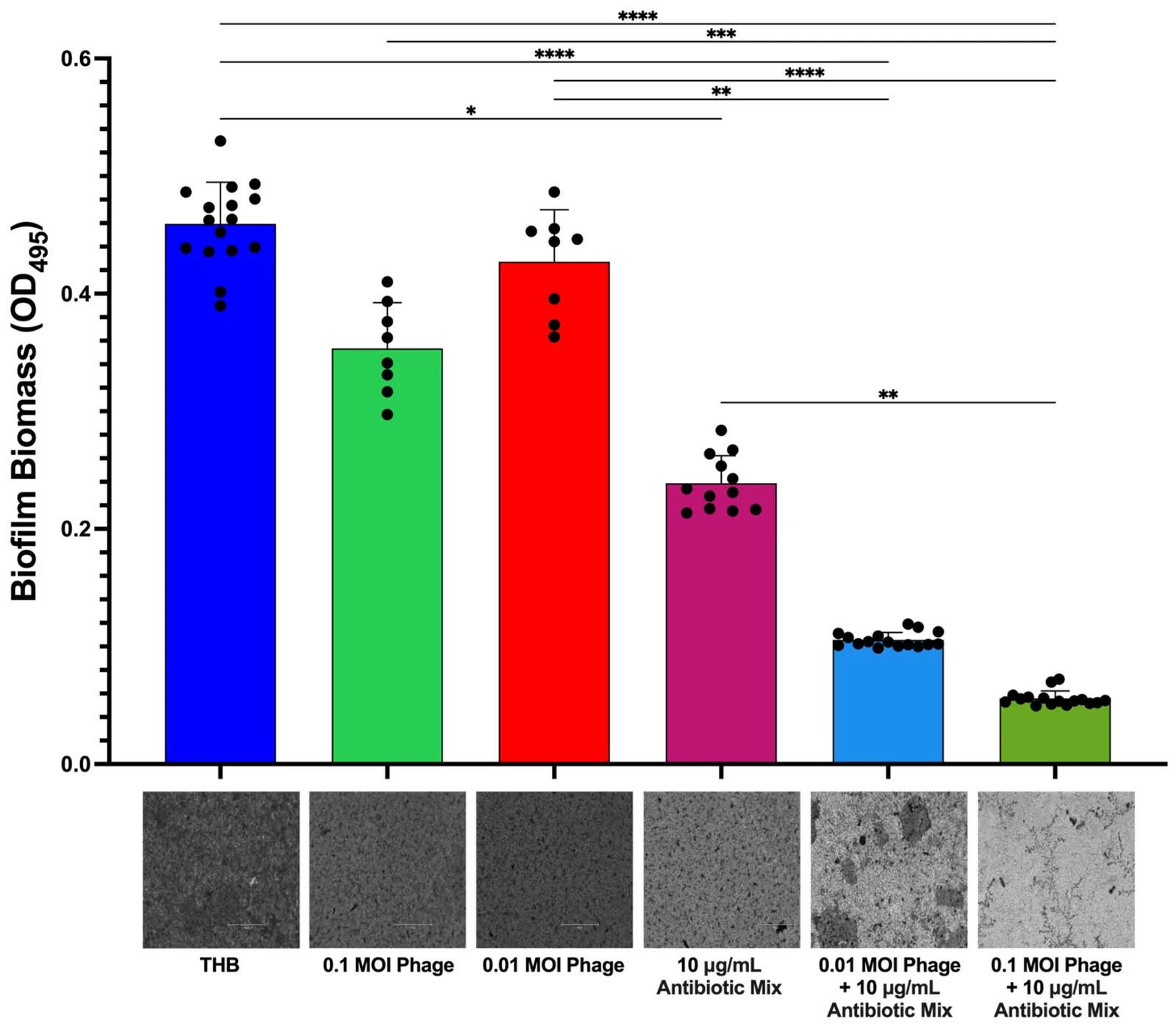
Antibiotic–bacteriophage combination has a synergistic effect against *S. marcescens* biofilms. Biofilm biomass quantification following *S. marcescens* UT-383 treatment with the antibiotic cocktail totaling 10 µg/mL, phage at MOI 0.1 or 0.01, or combinations at the same doses. Crystal violet staining was used to assess biofilm biomass post-treatment. Statistical differences were determined by the Kruskal–Wallis test with Dunn’s multiple-comparison post-test. * *p* ≤ 0.05, ** *p* ≤ 0.01, *** *p* ≤ 0.001, **** *p* ≤ 0.0001 (*n* = 8). Error bars denote standard deviation.

To determine the efficacy of these phages on mature *S. marcescens* biofilms, we tested each phage at MOIs of 0.01, 0.1, 1, and 10. We observed that ϕJCVI_Sm2-challenged biofilms were slightly more efficient in reducing biofilm biomass than ϕJCVI_Sm1 at lower MOIs (Supplemental Figure S1E). When we tested ϕJCVI_Sm2 alone and in combination with our antibiotic mixture, the results revealed that the combination of an MOI of 0.1 phage with a 10 µg/mL antibiotic mixture reduced biofilm biomass by 88%, compared to a 63% reduction achieved by the antibiotic mixture alone (Figure 4). Microscopy confirmed that biofilm architecture was severely disrupted in combined treatments, while completely intact biofilm architecture was only present within the single treatments. These results highlight the synergistic effects of phage therapy and antibiotic combinations on biofilm disruption.

### 2.5. MDR S. Marcescens Isolates Have Differential Responses to Phage–Antibiotic Treatment

**Figure 5:**
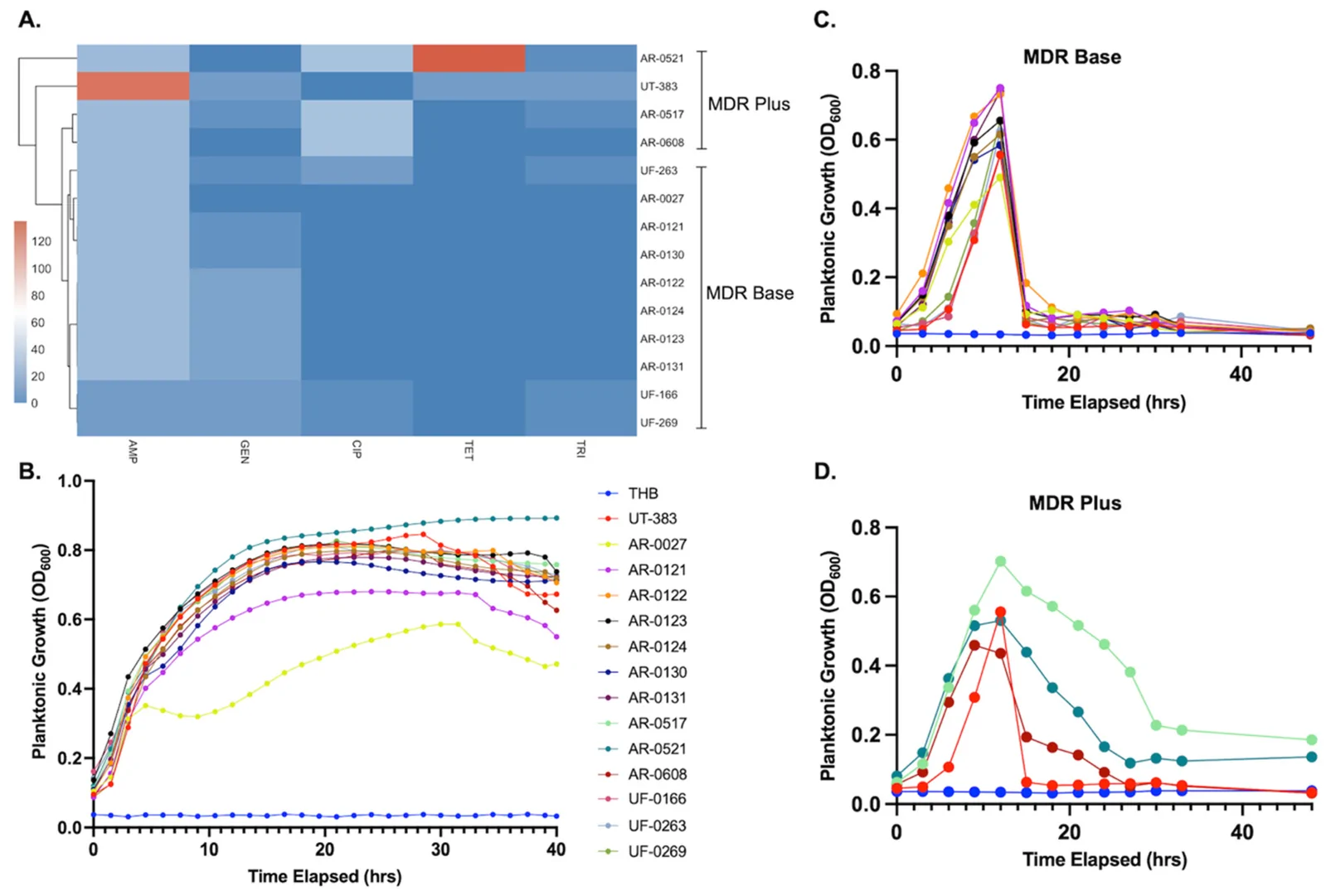
Phage–antibiotic combination therapy reduces growth of multidrug-resistant planktonic *S. marcescens* isolates with differential resistance profiles. (**A**). Heat map comparing the minimum inhibitory concentrations (MICs) of various antibiotics across the 10 multidrug-resistant *Serratia marcescens* strains obtained from the CDC as well as 3 strains isolated from Maryland farms. (**B**). OD_600_ planktonic growth curve data for the 14 tested strains measured over 40 h without antibiotics (*n* = 12). (**C**). Growth curve conducted with the MDR Base strains from panel A challenged with the phage–antibiotic cocktail (*n* = 12). (**D**). Growth curve conducted with the MDR Plus strains from panel A, challenged with the phage-antibiotic cocktail (*n* = 12). Different colors denote different strains to allow for easier identification.

Since the phage–antibiotic treatment was only tested on a single bacterial isolate, we aimed to test the efficacy of our phage–antibiotic cocktail on a panel of MDR strains of *S. marcescens*. The panel was composed of UT-383[30], ten strains obtained from clinical isolates from the CDC Antimicrobial Resistance Isolate Bank [31], and three isolates obtained from the University of Maryland from soil or plant rhizosphere samples from regional farms [32]. The MIC and susceptibility patterns for ampicillin, gentamicin, tetracycline, ciprofloxacin, and trimethoprim for each isolate are shown in Table S2. We observed that several of the CDC isolates (i.e., AR0517, AR0521, and AR0608), along with UT-383, had a broader resistance profile than all other tested isolates. When comparing the differential susceptibility profiles of ampicillin, gentamicin, tetracycline, ciprofloxacin, and trimethoprim across all 14 *S. marcescens* isolates using unsupervised hierarchical clustering, we observed that there were two different clusters defined by MIC (Table S2, Figure 5A). Hierarchical clustering of the MIC profiles separated the isolates into two study-specific groups. The “MDR Plus” group consisted of UT-383, AR0517, AR0521, and AR0608, which displayed the broadest resistance profiles and higher MICs across the antibiotics tested, whereas the remaining MDR isolates were designated “MDR Base” based on their comparatively lower resistance profiles. These designations were used to describe relative differences within our isolate panel and do not represent standardized MDR classifications. To establish a baseline for the growth kinetics of all 14 isolates, we generated growth curves for each isolate for up to 40 h (Figure 5B). The data revealed that most of the isolates had a similar growth rate except for AR0027, which showed lower logarithmic growth in THB medium (Figure 5B). When testing the activity of our phage–antibiotic cocktail on the isolates, we challenged planktonic cultures after 12 h in late log phase with 10 µg/mL of the antibiotic mixture combined with phage at an MOI of 0.1 (Figure 5C,D). The MDR Base isolates showed high susceptibility to the phage–antibiotic cocktail with a maintained reduction in absorbance three hours post-treatment. However, the MDR Plus isolates showed tolerance to the phage–antibiotic cocktail. These observations underscore the effectiveness and limitations of phage–antibiotic mixtures in overcoming MDR.

### 2.6. A Triple Combination of Antibiotics, Bacteriophages, and Antimicrobial Peptides Substantially Reduces Biofilm Biomass and Overcomes Resistance in Tolerant Strains

**Figure 6:**
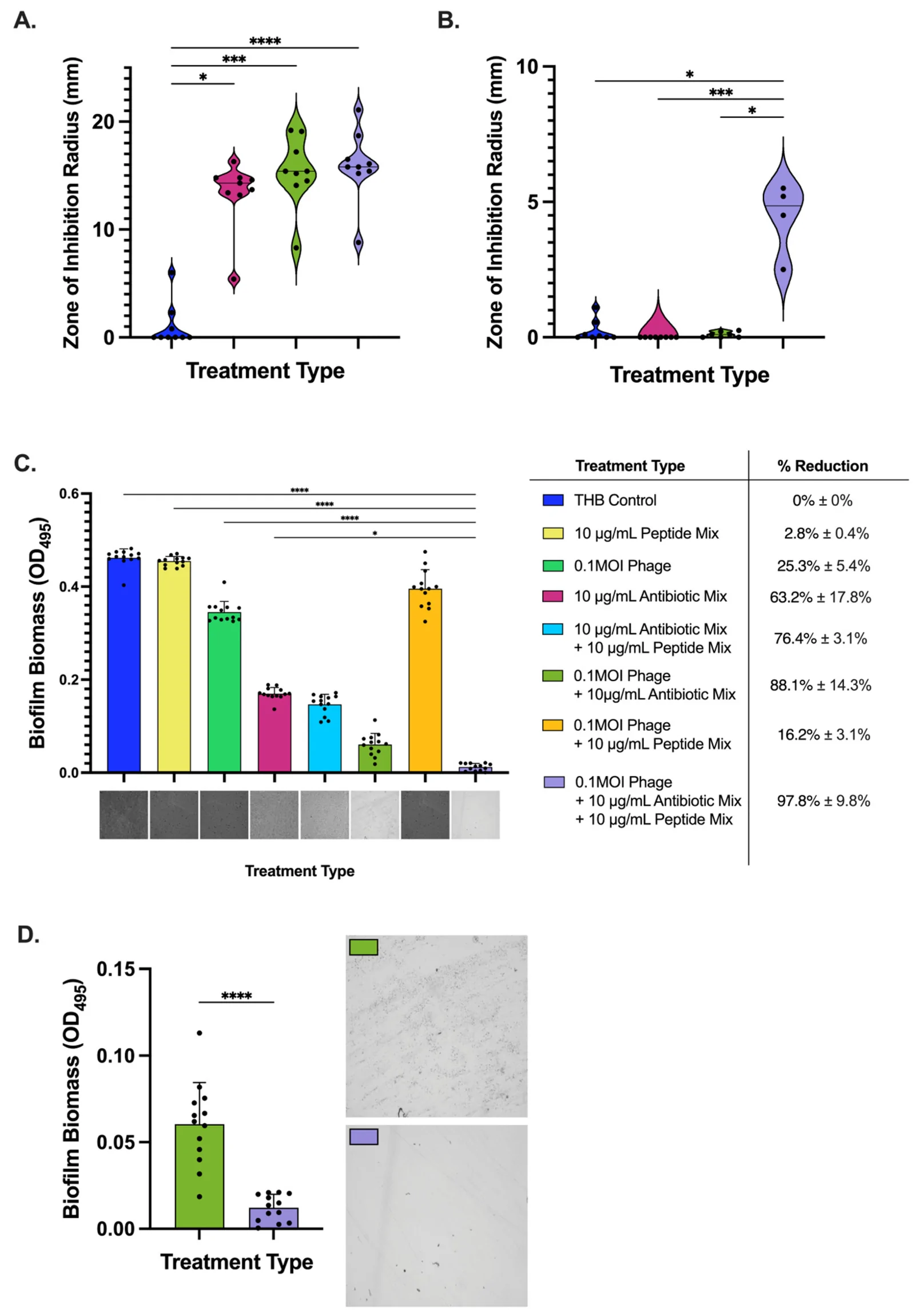
Synergistic combination of BAP achieves complete decolonization of *S. marcescens* biofilms. (**A**). Zone of inhibition analysis using disk diffusion assays of bacteriophages ϕJCVI_SM1 + ϕJCVI_SM2, antibiotics, and peptides in all permutations against S. marcescens bacterial lawns of *S. marcescens* MDR isolates, or (**B**). *S. marcescens* MDR-plus isolates. (**C**). Biofilm biomass quantification after crystal violet staining (490 nm) of *S. marcescens* following treatment with antibiotics, bacteriophage, peptides, and permutations thereof. (**D**). Biofilm biomass of the Phage + antibiotics mix compared to the BAP cocktail. Statistical differences were determined by the Kruskal–Wallis test with Dunn’s multiple-comparison post-test. * *p* ≤ 0.05, *** *p* ≤ 0.001, **** *p* ≤ 0.0001 (*n* = 12). Error bars denote standard deviation.

Since the combination of phage and antibiotics was insufficient to eradicate all strains of *S. marcescens* tested, we questioned whether the integration of antimicrobial peptides (AMPs) active against a wide range of Enterobacterales and previously isolated by our group from *Lentilactobacillus hilgardii* [33], into our phage–antibiotic cocktail could enhance treatment efficacy against mature *S. marcescens* biofilms. Mechanistically, this combined BAP approach leverages the combined actions of penicillin–streptomycin disrupting peptidoglycan synthesis and protein translation, kanamycin halting protein production via 30S ribosomal binding, ciprofloxacin impairing DNA gyrase and topoisomerase IV, AMPs presumably compromising membrane integrity through pore formation, and bacteriophages hijacking bacterial replication and/or inducing lysis. Disk diffusion assays demonstrated that in MDR Base strains, the BAP treatment produced the largest inhibition zones, marginally surpassing the phage–antibiotic combination and exceeding antibiotics alone (Figure 6A). In MDR Plus isolates, inhibition zones were significantly reduced across single and double treatments. However, the BAP therapy achieved a mean zone of 4.43 mm, a 15.73-fold increase over the THB control (Figure 6B), demonstrating enhanced growth inhibition under the conditions of the disk diffusion assay. Because phages, antibiotics, and peptides have different diffusion and replication properties, inhibition radius was interpreted as a comparative screening measure rather than a direct quantitative measure of therapeutic potency.

**Figure 7:**
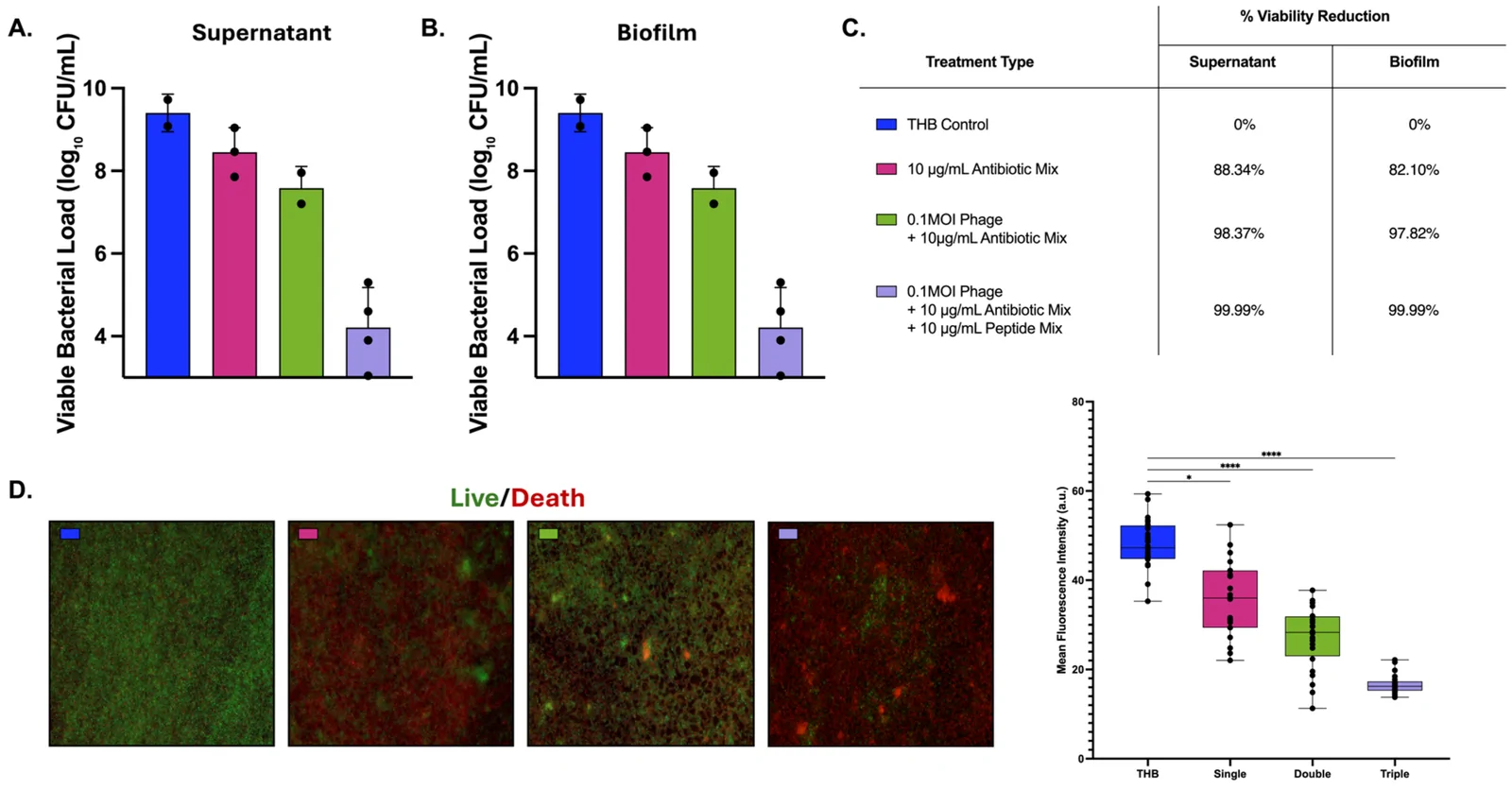
Synergistic combination of BAP achieves substantial reduction of viable *S. marcescens* biofilms. CFU of *S. marcescens* biofilm (**A**). Supernatant (*n* = 12) or (**B**). plate-bound bacteria (n = 12) after treatment with the antibiotic mix, the phage + antibiotic mix, and the BAP cocktail. (**C**). Normalized data analysis of CFU reductions. (**D**). Live/death fluorescent staining and mean fluorescent intensity measurement for the Live (green) and Death (red) channels. Statistical differences were determined by the Kruskal–Wallis test with Dunn’s multiple-comparison post-test. * *p* ≤ 0.05, **** *p* ≤ 0.0001. Error bars denote standard deviation (*n* = 18).

Next, we tested BAP against mature biofilms of *S. marcescens* AR-0517, the CDC isolate with the highest observed antibiotic resistance profile. Absorbance quantification confirmed that the triple antimicrobial treatment reduced biomass by 97.8% relative to the untreated control (Figure 6C,D). This surpassed the reductions observed with antibiotics alone (63.2%), phage + antibiotics (88.1%), antibiotics + peptides (76.4%), and phage + peptides (16.2%) permutations, while the peptides alone showed negligible anti-biofilm activity (Figure 6C). Microscopic evaluation of mature *S. marcescens* biofilms corroborated these findings, demonstrating extensive disruption of biofilm architecture following BAP treatment (Figure 6C,D). Viability assays confirmed that biomass reductions corresponded to bacterial killing, with BAP treatment producing a substantial log reduction in CFU/mL in both supernatant and biofilm fractions compared with untreated controls, while no significant regrowth was observed (Figure 7A–C). To support these results, we stained BAP-treated and untreated biofilms with a live–dead stain and observed that there was a stark reduction in green (live) fluorescence in the treated biofilms, with BAP treatment being the most effective. Mean fluorescence intensity (MFI) analyses of live-stained cells revealed a stepwise decline with treatment intensification (Figure 7D). To quantitatively evaluate interactions among treatments, Bliss independence analysis provided evidence of synergistic activity at the tested concentrations, with the phage–antibiotic and triple combinations producing positive ΔBliss values of +14.3 and +24.7 percentage points, respectively, both remaining significant after Holm correction (adjusted *p* = 0.00665); the antibiotic–peptide combination also showed a smaller but significant positive ΔBliss of +4.47 percentage points (adjusted *p* = 0.00833). In contrast, the phage–peptide combination produced a negative ΔBliss of −11.6 percentage points (adjusted *p* = 0.00833), indicating an antagonistic interaction in the absence of antibiotics and further supporting the importance of the antibiotic component in the enhanced activity of the BAP treatment (Supplemental Figure S2). Of note, a subtraction experiment showed that out of the combined antibiotics (kanamycin, penicillin–streptomycin, or ciprofloxacin), ciprofloxacin was the major antibiotic effector of the triple modality treatment, supporting the greatest decolonization (Supplemental Figure S3). Together, these data confirm that biomass loss corresponds to reductions in bacterial viability, not just matrix disruption and well clearance.

Finally, *S. marcescens* is known to cause severe infections associated with the respiratory tract [30,34,35]. We therefore evaluated the toxicity of BAP in human airway epithelial cells by means of a lactate dehydrogenase (LDH) assay. No detectable LDH release was observed after 12 or 24 h of treatment (Supplemental Figure S4), supporting initial cytocompatibility of the BAP combination under the conditions tested. However, this assay represents a preliminary assessment and does not constitute a comprehensive evaluation of cellular toxicity. Collectively, these results demonstrate that the triple antimicrobial BAP combination strategy effectively breaks through *S. marcescens* biofilm defense and resistance mechanisms, achieving superior biofilm disruption and bacterial clearance in MDR strains without harming mammalian cells.

## 3. Discussion

The management of *S. marcescens* infections continues to be hindered by its intrinsic and biofilm-associated resistance to a wide range of antibiotics. Here, we demonstrate that multidrug-resistant *S. marcescens* sourced from various environments exhibits variable susceptibility to distinct antibiotic classes depending on its physical state, as planktonic cells or in biofilm. Initial screening using antibiotics that target major bacterial processes, including cell wall synthesis (β-lactams), protein synthesis (aminoglycosides, tetracyclines, chloramphenicol), DNA replication (fluoroquinolones), and folate metabolism (sulfonamides), revealed significant differences in their efficacy, which may be due to *S. marcescens’s* broad genomic diversity [36]. While several agents, such as penicillin–streptomycin, ciprofloxacin, and kanamycin, substantially inhibited planktonic bacteria at high concentrations, their effects as single therapeutics were markedly diminished against mature biofilms. Among the tested agents, penicillin–streptomycin, kanamycin, and ciprofloxacin retained some biofilm-disruptive capacity, while others like gentamicin and trimethoprim showed only partial effects. These results highlight the limitations of monotherapy and underscore the therapeutic challenge posed by biofilm-associated infections [17]. Importantly, we showed that combining antibiotics targeting distinct cellular pathways enhanced clearance of both planktonic and biofilm-associated *S. marcescens*, and that this effect was further amplified when bacteriophages and antimicrobial peptides were added. This multi-tier antimicrobial combination reduced recoverable bacteria within mature *S. marcescens* biofilms by >99.99%. Together, these findings emphasize the importance of multidimensional antimicrobial strategies for overcoming the resistance of *S. marcescens* biofilms in community and nosocomial environments.

For *S. marcescens* and other nosocomial isolates, susceptibility is generally highest for aminoglycosides (e.g., gentamicin, amikacin), third-generation cephalosporins (e.g., cefotaxime), fluoroquinolones (e.g., ciprofloxacin, levofloxacin), and trimethoprim–sulfamethoxazole (cotrimoxazole), although resistance trends are escalating [37,38]. Intrinsic resistance stems from chromosomally encoded AmpC β-lactamases and efflux pumps like SdeAB, which confer resistance to penicillins, first- and second-generation cephalosporins, chloramphenicol, fluoroquinolones, and tetracyclines [39]. Acquired resistance occurs most commonly through plasmid horizontal gene transfer or through genetic mutations [7]. In the context of biofilms, high-dose antibiotic lock therapy with chloramphenicol has demonstrated significant biofilm biomass reduction and bacterial viability suppression on catheter-like surfaces in vitro [8]. However, chloramphenicol efficacy can be limited by efflux pump-mediated resistance pathways [40]. Similarly, combination strategies using antibiotic-loaded mesoporous silica nanoparticles (MSNs) have shown near-complete eradication of biofilms when antibiotics such as levofloxacin or gentamicin are stably released over prolonged periods, although direct application to *S. marcescens* remains to be tested [41]. Thus, to date, knowledge remains limited regarding how new antibiotic mono- or combinatorial strategies may be effective against *S. marcescens* biofilms.

Bacteriophages have emerged as promising therapeutic alternatives for combating AMR pathogens, including *S. marcescens*. Phage therapy offers pathogen-specific lytic activity, the potential to penetrate biofilms, and minimal impact on commensal microbiota, making it a particularly attractive option for multidrug-resistant *S. marcescens* infections. Studies have identified and characterized lytic phages active against clinical *S. marcescens* isolates, demonstrating differential efficacy in both planktonic and biofilm-associated states [42,43]. Moreover, phage–antibiotic synergy has been observed for other antimicrobial-resistant pathogens such as *Acinetobacter baumannii* and *Pseudomonas aeruginosa*, suggesting that combination therapy could enhance bacterial clearance and prevent resistance development [44]. However, no such study has been conducted for *S. marcescens* biofilms.

As resistance to conventional antibiotics continues to rise, particularly among nosocomial pathogens such as *S. marcescens*, multi-tiered antimicrobial interventions warrant further investigation in preclinical models. We also show that in addition to antibiotics and bacteriophages, the integration of antimicrobial peptides provided a synergistic approach to promote near-complete biofilm decolonization. Antimicrobial peptides are short, positively charged, amphipathic molecules that can have bactericidal activity mainly through membrane permeabilization [45]. Other mechanisms associated with AMPs are the targeting of intracellular bacterial processes and host immune modulation [45]. When combined with bacteriophages and/or conventional antibiotics, AMPs can promote the reduction of antibiotic resistance and lead to synergistic killing effects [46,47]. Thus, our results provide proof-of-concept support for further investigation of multi-tier antimicrobial strategies against *S. marcescens* biofilms and potentially other nosocomial pathogens. While static biofilms do not fully reproduce the flow, host factors, and nutrient conditions of device-associated infections, the robust activity observed here supports future evaluation of BAP in more clinically representative models.

The enhanced activity of the BAP combination likely reflects the simultaneous application of antimicrobial pressures with distinct biological targets, which may reduce the ability of *S. marcescens* biofilms to tolerate any single treatment modality. Importantly, treatment responses remained strain-dependent, with isolates exhibiting broader resistance profiles showing greater tolerance to phage–antibiotic treatment, while the addition of peptides increased activity even against the more resistant isolates. This is consistent with previous studies demonstrating enhanced antimicrobial effects when bacteriophages are combined with antibiotics or antimicrobial peptides, while extending this concept to a multimodal approach against *S. marcescens* biofilms.

In conclusion, the multifactorial nature of bacterial antimicrobial resistance requires experimental strategies capable of targeting multiple defense mechanisms simultaneously. Our findings provide in vitro proof-of-concept for the BAP approach and support its further evaluation in more clinically representative models. Future studies should assess its efficacy and safety in advanced in vitro and in vivo infection models and further define the mechanisms underlying the observed synergistic activity.

## 4. Methods

### 4.1. Planktonic Growth and Quantification

*Serratia marcescens* cultures were streaked onto blood agar plates from prepared stock and incubated overnight. A single colony was grown in 4 mL Todd Hewitt Broth (THB) (Sigma–Aldrich, St. Louis, MO, USA) at 37 °C for 24 h until the culture reached an optical density of 1.24 (OD_600_), equivalent to ~10^8^ CFU/mL. After 24 h of incubation, the culture was diluted with 4 mL of fresh THB medium to a total of 8 mL, to an approximate OD_600_ of 0.6, to start the growth curve. Growth measurements were recorded spectrophotometrically at OD_600_ every 30 min for 48 h. Planktonic growth was measured exclusively at OD_600_ with a limit of detection of 0.02 OD_600_. For the untreated 14-strain baseline growth curves shown in Figure 5B, OD_600_ was measured every 30 min for 40 h. For antimicrobial-treated planktonic growth curves shown in Figure 5C,D, OD_600_ was measured every 30 min for 48 h. Endpoint planktonic antibiotic-screening experiments shown in Figure 1 and Figure 3A were measured at OD_600_ after 24 h of treatment. OD_490_ was used only for crystal violet quantification of biofilm biomass as described in Section 4.2.

### 4.2. Biofilm Growth, Staining and Quantification

Biofilms were established by streaking *S. marcescens* liquid cultures onto blood agar plates and incubating them at 37 °C overnight to obtain isolated single colonies. A single colony was inoculated into 4 mL of THB medium (Sigma–Aldrich, USA) and incubated at 37 °C for 4–8 h. After reaching an OD_600_ of 1.20, cultures were diluted by adding 12 mL of fresh THB medium and transferred to well plates in the following volumes: 1 mL total (200 µL culture + 800 µL THB) for 24-well plates, 500 µL total (100 µL culture + 400 µL THB) for 48-well plates, and 250 µL total (50 µL culture + 200 µL THB) for 96-well plates. Plates were incubated statically at 37 °C for 24 h to allow biofilm formation. At 24 h, the medium was gently aspirated and replaced with fresh THB medium before incubating the plates for an additional 24 h. To quantify the biofilms, the medium was removed, and the biofilms were washed 3 times with sterile phosphate-buffered saline (PBS) (Gibco, Grand Island, NY, USA, pH 7.4), air-dried, and then fixed with 4% formaldehyde (Sigma–Aldrich, USA) for 10 min. After fixation, formaldehyde was removed, and biofilms were stained with 1% crystal violet for 10–15 min. Excess stain was removed, and wells were washed three times with PBS. Biofilms were then air-dried for 5–10 min before imaging on a Leica DMi1 microscope. To quantify biofilm biomass, 200 µL of 70% ethanol was added to each well to extract the crystal violet stain, and absorbance was measured at OD_490_ using a Varioskan Lux microplate reader (Thermo Fisher Scientific, Waltham, MA, USA).

### 4.3. Phage Wastewater Processing

Bacteriophages were isolated from untreated inflow wastewater obtained from a local water treatment facility in Poolesville, MD, USA. Samples were first centrifuged in 100 mL aliquots for 30 min at 7500× *g* at 4 °C to pellet solids and sediment. Supernatants were then vacuum-filtered through a 0.2 µm filter to remove bacteria and stored for future experiments.

**Phage Enrichment.** *S. marcescens* bacterial isolates UT-383 [30] and HER1170 [48] were inoculated in Tryptic Soy broth (TSB) (Sigma–Aldrich, USA) and grown overnight at 37 °C with shaking. The next day, 20 mL of fresh TSB, 5 mL of processed wastewater, and 50 µL of overnight host bacterial culture were mixed in a 150 mL Erlenmeyer flask and again incubated overnight at 37 °C with shaking. The next day, “enriched” cultures were transferred to 50 mL conical centrifuge tubes and spun at 8000× *g* for 15 min to pellet bacterial cells. Supernatants were then vacuum-filtered through a 0.2 µm filter to remove bacteria.

### 4.4. Isolation of Bacteriophages

Bacteriophages were identified using a soft agar overlay assay. Briefly, 150 mm bottom agar Petri plates were prepared containing TSB with 1.5% agar, 1 mM CaCl_2,_ and 1 mM MgCl_2_. *S. marcescens* bacterial isolates MB383 and HER1170 were inoculated into TSB and grown overnight at 37 °C. A volume of 300 µL of stationary-phase culture and 300 µL of “enriched” wastewater were added to a tube and incubated at room temp for 10 min. Then, 10 mL of top agar (TSB with 0.7% agarose, 1 mM CaCl_2_, and 1 mM MgCl_2_) cooled to 55 °C was added, and the mixture was plated onto a bottom agar plate and solidified. Plates containing bacterial culture and top agar only were used as a negative control. Plates were incubated overnight at 37 °C and were examined the following day to identify lytic phage plaques. Phages were passaged by sequential picking and plating of individual plaques grown on the same bacterial isolate. Phages were picked from individual plaques using a pipette tip and were placed into 100 μL of SM buffer (50 mM Tris-HCl, pH 7.5, 100 mM NaCl, 8 mM MgSO_4_) and incubated overnight at 37 °C. The following day, 10-fold serial dilutions were made in SM buffer, and 10 μL of each dilution was spotted onto a plate containing 5 mL of top agarose mixed with 100 μL of stationary-phase bacterial culture and layered on top of bottom agar. After overnight incubation at 37 °C, an individual plaque was picked and passaged again. Each phage was passaged three times before the generation of high-titer stocks.

### 4.5. Phage Stock Generation

To generate high-titer stocks, a single plaque was picked into 100 μL of SM buffer and incubated overnight. Then, 100 μL of overnight bacterial culture (OD_600_ > 1.0) was added, and the mixture was incubated for 5–10 min at room temperature, followed by the addition of 10 mL of top agarose and plating onto large bottom agar plates. Plates were incubated overnight at 37 °C, and then plates with high plaque density were flooded with 10 mL of SM buffer and incubated at 37 °C for at least 1 h to elute phages from the top agar. The SM buffer was removed from each plate, pooled, spun down at 4,000 rpm for 20 min, and filtered through a 0.2 μm filter, titered, and stored at 4 °C.

### 4.6. Phage DNA Isolation and Whole Genome Sequencing

Bacteriophage DNA was extracted from liquid lysate stocks using the NORGEN Phage DNA Isolation Kit (Norgen Biotek Corp., Thorold, ON, Canada) according to the manufacturer’s instructions. Whole-genome sequencing was performed by SeqCoast Genomics (Portsmouth, NH, USA) on an Illumina NextSeq2000 using an nXLEAP-SBS 300-cycle flow cell, producing 2 × 150 bp reads. Sequence reads were demultiplexed, trimmed, and quality-analyzed using DRAGEN v4.2.7 software. The remaining reads were downsampled using Reformat from bbmap (https://sourceforge.net/projects/bbmap/, accessed on 8 March 2025), to produce between 140,000 and 150,000 paired-end reads. Downsampled reads were assembled into single genomic contigs using Unicycler v0.5.1 with default settings. Pharokka (PMID: 36453861) v1.8.2 was used to annotate the phage genomes. The NCBI BLASTN (PMID: 10890397) web server, optimized for highly similar sequences (i.e., megablast), was used to search the nr/nt nucleotide database to identify top phage matches using default settings. Linear genomic comparison figures were generated using LinearDisplay.pl (github.com/JCVenterInstitute/LinearDisplay), mapping coding sequence (CDS) similarity using BLASTP top matches with at least 20% protein sequence identity. Bacteriophage genome sequences are publicly available under NCBI Bioproject PRJNA1475042.

### 4.7. Peptide Synthesis

The bioinformatically predicted peptide sequences from *Lentilactobacillus hilgardii* previously published by our group were chemically synthesized by LifeTein LLC (Somerset, NJ, USA) to a minimum of 85% purity [33]. The two most effective peptides were combined in equal proportions to generate the peptide cocktail. A total peptide concentration of 10 μg/mL corresponded to approximately 5 μg/mL of each peptide.

### 4.8. Bacteriophage, Antibiotics and Peptides (BAP) Treatment

Both planktonic and biofilm cultures were treated with BAP. Antibiotic solutions were diluted in THB medium from their starting concentrations to the intended doses [8]. Penicillin–streptomycin was prepared as a 1:1 mixture of penicillin G sodium (Thermo Scientific Chemicals, Waltham, MA, USA, cat. J63032.22) and streptomycin sulfate (Gibco, Grand Island, NY, USA, cat. 11860038). At a total antibiotic cocktail concentration of 100 µg/mL, the final concentrations were 16.65 µg/mL penicillin G sodium, 16.65 µg/mL streptomycin sulfate, 33.3 µg/mL kanamycin, and 33.3 µg/mL ciprofloxacin. Accordingly, the 10 µg/mL cocktail used in the principal phage–antibiotic and BAP experiments contained 1.665 µg/mL penicillin G, 1.665 µg/mL streptomycin, 3.33 µg/mL kanamycin, and 3.33 µg/mL ciprofloxacin. Bacteriophage stocks were approximately 1 × 10^8^ PFU/mL. For mature biofilms, bacterial density at the time of treatment was estimated from the corresponding untreated growth curve using the OD600-to-cell-density relationship, and the required phage input was calculated from this estimate to achieve the indicated MOI. Bacteriophage ϕJCVI_Sm2 was used at an MOI of 0.1. Peptides were diluted from stock concentrations in molecular biology-grade water (Corning, Manassas, VA, USA) and combined in equal proportions to a final total peptide cocktail concentration of 10 μg/mL.

Planktonic cultures were grown as described above, and 250 µL of bacterial culture was added to each well of a 24-well plate. An additional 250 µL of treatment solution was then added for a final well volume of 500 µL. Treatment solutions were prepared at twice the intended final concentration so that, after mixing with the bacterial culture, all reported concentrations represent the final concentration in the well. Plates were incubated at 37 °C with shaking at 120 rpm, and OD600 measurements were recorded every 30 min for 48 h to assess treatment effects on planktonic growth dynamics.

For biofilm experiments, cultures were grown as described above until immediately prior to fixation and quantification. The existing medium was aspirated, and each biofilm received 250 µL of fresh THB and 250 µL of treatment solution for a final well volume of 500 µL. As in the planktonic experiments, treatment solutions were prepared such that the reported antibiotic and peptide concentrations and bacteriophage MOIs represent the final concentrations in the well after mixing. Antibiotics were used at the indicated final concentrations, the peptide cocktail was used at a final total concentration of 10 µg/mL, and bacteriophage input was adjusted to achieve the indicated final MOI. Biofilms were incubated for an additional 24 h at 37 °C without shaking. Following treatment, the medium was aspirated, and the biofilms were washed with sterile PBS before staining, imaging, and quantification as described above.

### 4.9. Disk Diffusion Assay

To evaluate the zone of inhibition induced by different treatment combinations, a disk diffusion assay was performed using all 14 *S. marcescens* strains described in this study. Each strain was cultured in 4 mL Todd Hewitt Broth (THB) at 37 °C for 24 h until the culture reached an optical density around 1.3 (OD_600_), equivalent to ~10^8^ CFU/mL. Then, 100 μL of the solution was uniformly spread using a sterile cotton swab to ensure even bacterial lawn formation. For each strain, the plate was divided into 4 quadrants, and four sterile paper disks (Microxpress, Verna, India) were placed equidistantly, one in each quadrant on the agar surface. The first disk served as a negative control, containing only Todd Hewitt Broth (THB) without any antimicrobial agents. The second disk contained the cocktail antibiotic solution at 100 µg/mL, composed of penicillin–streptomycin, kanamycin, and ciprofloxacin. The third disk consisted of the same antibiotic combination supplemented with bacteriophage at an MOI of 0.1. The fourth disk contained the full treatment, which consisted of the antibiotic cocktail, bacteriophage (MOI 0.1), and 10 µg/mL of AMPs from *L. hilgardii*. Plates were incubated at 37 °C for 24 h, after which the inhibition radius was measured from the outer edge of the 10 mm-diameter disk to the outer boundary of visible growth inhibition using a ruler; the diameter of the disk itself was not included in the measurement. These measurements allowed for a comparative assessment of antimicrobial efficacy across treatment types and strains, with larger zones indicating higher levels of bacterial growth inhibition.

### 4.10. Minimum Inhibitory Concentration Assay

An overnight culture of *S. marcescens* was started and was diluted 1:10,000 the next day in fresh THB medium. All antibiotics used were sterile-filtered for this assay and used at the indicated concentrations. First, serial two-fold dilutions of antibiotics were prepared starting at the highest concentration. Then, 100 µL of antibiotic at the indicated concentration was mixed with 100 µL of diluted *S. marcescens* culture in each well of a 96-well plate, in triplicate. The plate was then incubated at 37 °C overnight and was observed the next day for the lowest concentration of antibiotic that inhibited bacterial growth. This concentration was determined to be the minimum inhibitory concentration. Isolates were classified as multidrug-resistant (MDR) according to accepted international criteria, with MDR defined as non-susceptibility to at least one antimicrobial agent in three or more antimicrobial categories. The terms “MDR Base” and “MDR Plus” were used only as study-specific designations to distinguish isolates with relatively lower versus broader MIC resistance profiles among the antibiotics tested and do not represent standardized resistance categories.

### 4.11. Colony-Forming Unit Viability Assay

Colony-forming unit (CFU) viability assays were performed following BAP treatment to assess bactericidal activity against *S. marcescens*. Prior to CFU enumeration, treated samples were washed three times with sterile PBS to minimize antimicrobial carryover from residual antibiotics, bacteriophages, and peptides. Cultures were separated into supernatant and biofilm-associated fractions. Biofilm-associated cells were recovered into 1 mL of sterile PBS by scraping, followed by repeated pipetting and vortexing to disrupt and homogenize the biofilm. Supernatant and recovered biofilm fractions were serially diluted in sterile PBS, and 10 µL of each dilution was spot-plated onto blood agar in triplicate. Plates were incubated at 37 °C for approximately 16 h before colonies were enumerated and CFU/mL was calculated. Based on the 10 µL spot-plating volume, the nominal limit of detection was 100 CFU/mL (2 log10 CFU/mL). Samples with no recoverable colonies were reported as below the limit of detection rather than as sterile.

### 4.12. Mean Fluorescence Intensity Imaging

*S. marcescens* biofilms were grown overnight in 200 µL of THB at 37 °C in an 8-well chamber removable slide (Ibidi, Gräfelfing, Germany, cat. 80841), then exposed for 24 h to THB alone (control) or to our Single, Double, or Triple treatment. After aspiration of the supernatant, biofilms were stained using the LIVE/DEAD BacLight Bacterial Viability kit (Invitrogen, Carlsbad, CA, USA) following the manufacturer’s protocol, with CFU enumeration used separately as the primary measure of bacterial culturability and viability. Fluorescence images were captured immediately on a Leica SP8 confocal microscope (40× objective). For each well, five non-overlapping Z-stacks (20 µm, 1 µm steps) were acquired and rendered as maximum-intensity projections. Mean fluorescence intensity for green (live) and red (dead) channels was quantified in Fiji after background subtraction and averaged across fields. Images included in the manuscript were processed in ImageJ (version 1.54r 25) to separate the fluorescent channels (red and green).

### 4.13. Lactate Dehydrogenase Release Assay

The lactate dehydrogenase (LDH) assay was used to evaluate the cytotoxicity of the tested combination of antibiotics, peptides, and phages in alveolar type II epithelial cells. (A549, ATCC CCL-185). Briefly, A549 cells were maintained in sterile-filtered DMEM supplemented with 10% FBS and 5% CO_2_. For the LDH assay, cells were seeded in 96-well plates at 5 × 10^4^ cells/well. The medium was replaced with RPMI 1640 containing 2% FBS and a cocktail of antibiotics, peptides, and phages at the experimentally tested concentrations. Plates were incubated for 12 or 24 h at 37 °C, 5% CO_2_. LDH release was quantified using the Invitrogen™ CyQUANT LDH Cytotoxicity Assay Kit per the manufacturer’s instructions. Absorbance was measured at OD_490_ and OD_680_ using the Varioskan LUX (Thermo Fisher, Waltham, MA, USA). Data analysis was conducted as described below. Experiments were performed in triplicate.

### 4.14. Statistical Analysis

Experiments were conducted using three independent biological replicates, with 4–8 technical replicates performed within each biological replicate, yielding a total of 12–24 data points per experimental condition. The number of biological and technical replicates (n) used for each experiment is also specified in the corresponding figure legend. Statistical tests were selected based on the structure of each dataset and the type of comparison being performed. For planktonic growth, biofilm biomass, CFU, and other antimicrobial treatment response experiments, non-parametric analyses were used to avoid reliance on assumptions of normality across treatment groups. Comparisons involving three or more treatment groups within a given strain or experimental condition were analyzed using the Kruskal–Wallis test followed by Dunn’s multiple-comparison post-test. For selected direct comparisons between two treatment groups, including comparisons intended to assess the effect of a specific treatment or treatment combination, Mann–Whitney U tests were performed. For experiments involving multiple factors, particularly treatment across multiple *S. marcescens* strains, separate direct statistical analyses were additionally performed within individual strains or predefined MDR groups rather than pooling all strains into a single comparison. This approach allowed treatment effects to be evaluated within each biological background while accounting for strain-to-strain differences in baseline growth and antimicrobial susceptibility. For the heat map representing antimicrobial susceptibility across strains, hierarchical cluster analysis was performed to group strains according to their resistance profiles and phenotypic similarities. Mean fluorescence intensity (MFI) measurements were analyzed separately for the live and dead fluorescence channels across treatment groups using one-way ANOVA followed by Tukey’s multiple-comparison post-test. Synergy among antimicrobial treatment combinations was quantitatively assessed using Bliss independence analysis [49]. Expected inhibition under independent action was calculated from the corresponding individual treatment effects and compared with the observed inhibition for each combination. The difference between observed and Bliss-predicted inhibition was expressed as ΔBliss, with positive values indicating activity greater than expected under independent action and negative values indicating activity below the Bliss expectation. Replicate-level ΔBliss values were compared with zero using Wilcoxon signed-rank tests, and *p*-values were adjusted for multiple comparisons using the Holm correction. Statistical analyses were performed using GraphPad Prism version 10.4.1 and R (version 4.6.1). Exact *p*-values are reported for the corresponding direct and Bliss comparisons where applicable. Statistical significance was defined as *p* < 0.05, with significance levels denoted as *p* < 0.05 (*), *p* < 0.01 (**), *p* < 0.001 (***), and *p* < 0.0001 (****).

## Data Availability

Bacteriophage genome sequences are publicly available under NCBI Bioproject PRJNA1475042.

## Supplementary Materials

The following supporting information can be downloaded at: https://www.mdpi.com/article/10.3390/antibiotics15090879/s1, Figure S1: Isolation and Effect of bacteriophages in *S. marcescens* soft-agar cultures and mature biofilms; Figure S2: Bliss independence analysis identifies synergistic interactions among antibiotic, phage, and peptide treatments against *S. marcescens* biofilms. Figure S3: Subtraction experiment shows ciprofloxacin as the major antibiotic effector of the triple modality treatment; Figure S4: Sub-MIC antibiotics, AMPs, and bacteriophage cocktail do not cause cytotoxicity in respiratory epithelial cells. Table S1: Bacterial targets of comma antibiotics. Table S2: MIC and susceptibilities of *Serratia marcescens* strains.
