# Supplementary material for: Phage–Antibiotic–Peptide Synergy Overcomes Biofilm-Mediated Multidrug Resistance in Serratia marcescens
Source: Antibiotics (Basel). 2026 Sep 8;15(9):879. doi: 10.3390/antibiotics15090879 (PMC13603727; doi:10.3390/antibiotics15090879)
Supplement: Supplementary file 1 [file antibiotics-15-00879-s001.zip › antibiotics-4527741-supplementary.pdf]

Supplemental Figures and Tables:

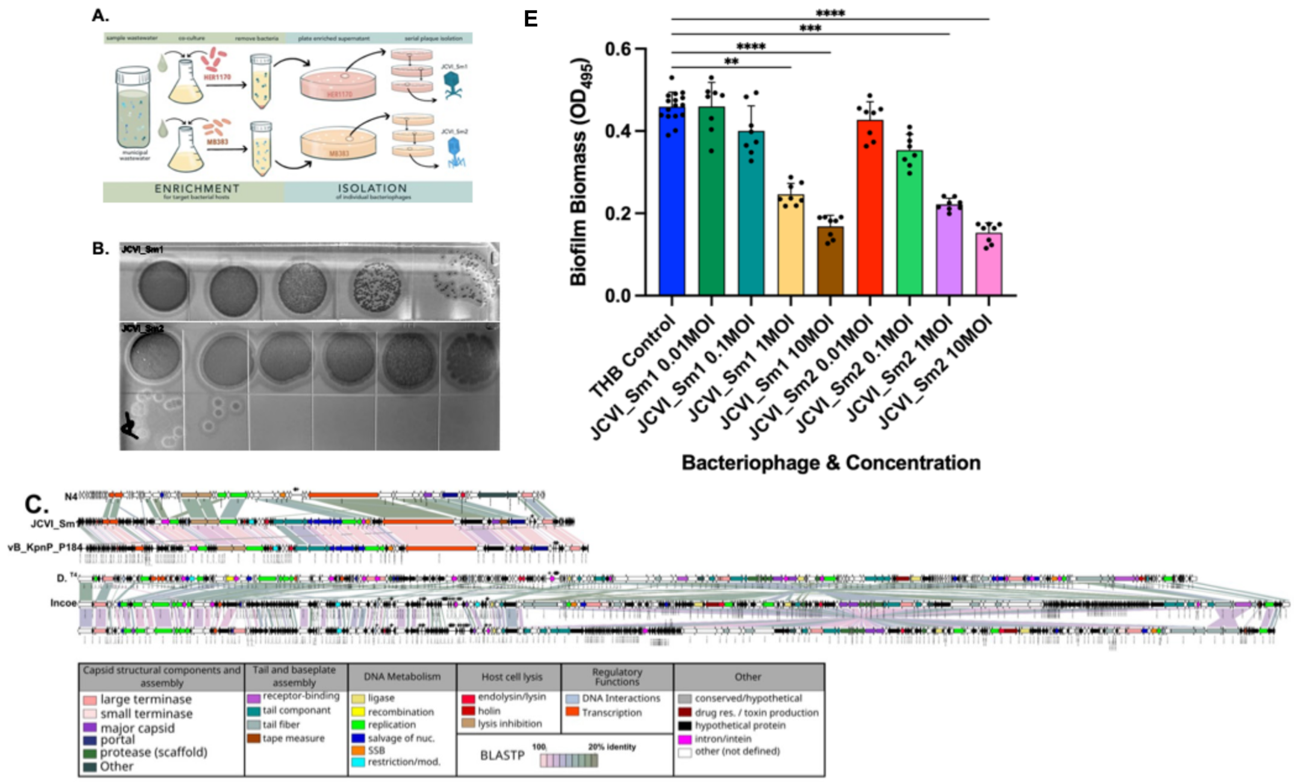

Figure S1. Isolation and Effect of bacteriophages in *S. marcescens* soft-agar cultures and mature biofilms.

A. Visual schematic of bacteriophage isolation from wastewater**. B.** Plaque assay for bacteriophages ϕJCVI_Sm1 and ϕJCVI_Sm2. **C.** Comparison of the genome of ϕJCVI_Sm1 (GenBank: PZ554734) to model coliphage N4 (GenBank: EF056009) and *Klebsiella* phage vB_KpnP_P184 (GenBank: NC_055919). **D.** Comparison of the genome of ϕJCVI_Sm2 (GenBank: PZ600306) to model coliphage T4 (GenBank: NC_000866) and *Serratia* phage Incoe (GenBank: PQ661788. Linear maps are based on nucleotide sequences of the phage genomes and predicted open reading frames. CDSs are labeled by locus identifier or gene symbol (for N4) and colored by functional role categories as noted in the boxed key. BLASTP matches between CDSs are colored by protein percent identity (see key). tRNAs are depicted by clover leaf structures. ϕJCVI_Sm2 and Incoe were rotated to align with the beginning of bacteriophage T4. The Incoe genome was rotated to position 112585, reverse complemented and reannotated with Pharokka version 1.8.2 (PMID: 36453861) using default settings to get functional annotations. The original locus_tags were mapped to the Pharokka annotation. Any new genes predicted by Pharokka were left unlabeled. **E.** *Serratia marcescens* UT-383 was grown in biofilm form for 48 hours prior to ϕJCVI_Sm1 or ϕJCVI_Sm2 at MOIs 0.01, 0.1, 1 and 10. Statistical differences were determined by the Kruskal–Wallis test with Dunn's multiple-comparison post-test. * $p \leq 0.05$, ** $p \leq 0.01$, *** $p \leq 0.001$, **** $p \leq 0.0001$. Error bars denote standard deviation.

A.

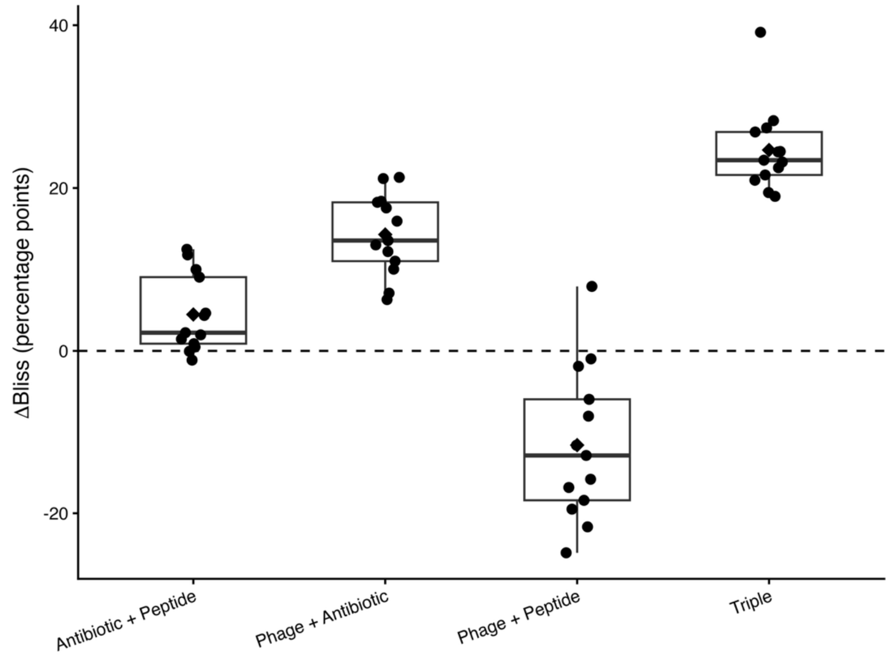

B.

Combination	Observed inhibition (%)	Bliss expected inhibition (%)	Delta Bliss	Wilcoxon p	Holm-adjusted p	Interpretation
**Antibiotic + Peptide**	68.2	63.73	4.47	0.00518	0.00833	Synergy relative to Bliss
**Phage + Antibiotic**	86.75	72.46	14.29	0.00166	0.00665	Synergy relative to Bliss
**Phage + Peptide**	14.1	25.67	-11.57	0.00417	0.00833	Antagonism relative to Bliss
**Triple**	97.38	72.72	24.67	0.00166	0.00665	Synergy relative to Bliss

Figure S2. Bliss independence analysis identifies synergistic interactions among antibiotic, phage, and peptide treatments against *S. marcescens* biofilms.

Biofilm biomass was measured by OD495 following treatment with antibiotic, phage, peptide, pairwise combinations, or the triple phage-antibiotic-peptide combination and normalized to the corresponding untreated THB control. A. Bliss excess (ΔBliss) was calculated for each replicate as the difference between observed inhibition and inhibition predicted under Bliss independence from prior literature. Positive ΔBliss values indicate synergy relative to the Bliss model, values near zero indicate independent/additive activity, and negative values indicate antagonism. Individual points represent biological replicates, diamonds indicate the mean, and boxplots show the median and interquartile range. B. Summary Table of observed inhibition, Bliss-expected inhibition, mean ΔBliss, and statistical comparisons for each treatment combination. Observed and Bliss-expected inhibition values were compared using paired Wilcoxon signed-rank tests with Holm correction for multiple comparisons. Antibiotic + peptide, phage + antibiotic, and the triple treatment demonstrated significant positive Bliss excess, whereas phage + peptide demonstrated significant antagonism relative to Bliss independence (n = 12).

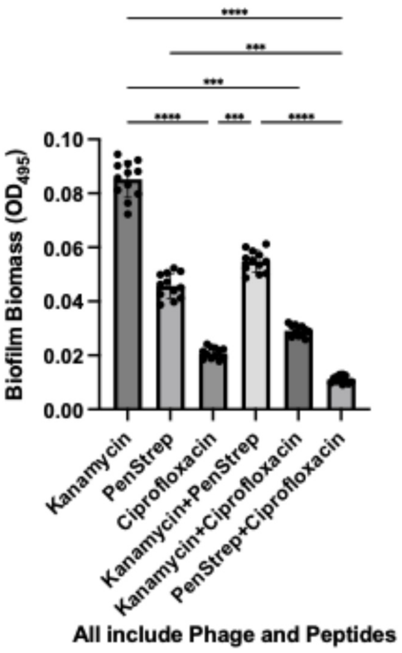

Figure S3. Subtraction experiment shows ciprofloxacin as the major antibiotic effector of the triple modality treatment.

Serratia marcescens UT-383 was grown in biofilm form for 48 hours prior to treatment with Phage + peptides with single or dual combination of Kanamycin, PenStrep, or Ciprofloxacin. Statistical differences were determined by the Kruskal–Wallis test with Dunn's multiple-comparison post-test. * $p \leq 0.05$, ** $p \leq 0.01$, *** $p \leq 0.001$, **** $p \leq 0.0001$. Error bars denote standard deviation.

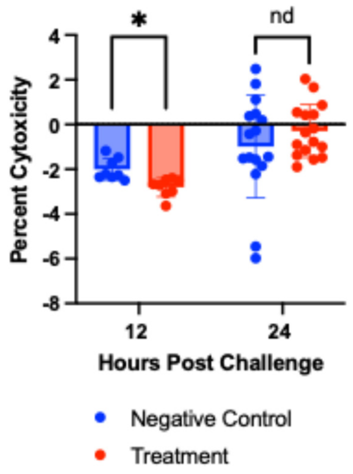

Figure S4. Sub-MIC antibiotics, AMPs, and bacteriophage cocktail do not cause cytotoxicity in respiratory epithelial cells.

A549 cells were challenged with the Sub-MIC antibiotics, AMPs and bacteriophage cocktail and supernatant was collected at 12 and 24 hour to assess release of lactate dehydrogenase. Statistical differences were determined by Two-way ANOVA. * $p \leq 0.05$, ** $p \leq 0.01$, *** $p \leq 0.001$, **** $p \leq 0.0001$. Error bars denote standard deviation.

Table S1. Bacterial targets of comma antibiotics.

Antibiotic	Classification	Mechanism of Action	Bacterial Component Targeted
**Cefotaxime**	Beta-lactam antibiotic (cephalo-sporin)	Inhibits cell wall synthesis	PBP on cell wall
**Trimethoprim**	Sulfonamide antibiotic	Inhibits folic acid synthe-sis	Dihydrofolate reductase
**Doxycycline**	Tetracycline antibiotic	Inhibits protein synthesis	30S ribosomal subunit
**Tetracycline**	Tetracycline antibiotic	Inhibits protein synthesis	30S ribosomal subunit
**Ampicillin**	Beta-lactam antibiotic (ami-nopenicillin)	Inhibits cell wall synthesis	Transpeptidase
**Chloramphenicol**	Broad spectrum antibiotic	Inhibits protein synthesis	50S ribosomal subunit
**Gentamicin**	Aminoglycoside	Inhibits protein synthesis	30S ribosomal subunit
**Ciprofloxacin**	Fluoroquinolone	Inhibits DNA replication	DNA gyrase & Topoisomerase IV
**Kanamycin A**	Aminoglycoside	Inhibits protein synthesis	30S ribosomal subunit
**Penstrep**	Beta-lactam & aminoglycoside	Inhibits cell wall	PBP on cell wall & 30S ribosomal sub-unit
**Co-trimoxazole**	Sulfonamide antibiotic	Inhibits folic acid synthe-sis	Dihydrofolate reductase & Dihydrop-teroate synthase

Table S2. MIC and susceptibilities of *Serratia marcescens* strains.

Isolate_ID	Organism Name	Ampicillin MIC	Gentamicin MIC	Tetracycline MIC	Ciprofloxacin MIC	Trimethoprim MIC
UT-383	Serratia marcescens	>32	16	16	0.5	16
AR-0027	Serratia marcescens	>32	≤0.25	16	1	2
AR-0091	Serratia marcescens	>32	1	4	≤0.25	≤0.5
AR-0099	Serratia marcescens	>32	0.5	8	0.5	≤0.5
AR-0121	Serratia marcescens	>32	0.5	8	≤0.25	≤0.5
AR-0122	Serratia marcescens	>32	0.5	8	≤0.12	≤0.5
AR-0123	Serratia marcescens	>32	0.5	8	≤0.12	≤0.25
AR-0124	Serratia marcescens	>32	0.5	8	≤0.12	0.5
AR-0130	Serratia marcescens	>32	1	>32	0.25	≤0.25
AR-0131	Serratia marcescens	>32	0.5	16	0.25	≤0.5
AR-0517	Serratia marcescens	>32	>16	>32	4	>8
AR-0520	Serratia marcescens	>32	8	8	2	>8
AR-0521	Serratia marcescens	>32	>16	>32	>8	>8
AR-0608	Serratia marcescens	>32	4	16	2	≤0.5
UF-166	Serratia marcescens	16	16	8	0.25	8
UF-263	Serratia marcescens	32	8	16	0.5	8
UF-269	Serratia marcescens	16	16	8	0.5	8

Isolate_ID	Organism Name	Ampicillin INT	Gentamicin INT	Tetracycline INT	Ciprofloxacin INT	Trimethoprim INT
UT-383	Serratia marcescens	R	R	R	S	R
AR-0027	Serratia marcescens	R	S	R	R	S
AR-0091	Serratia marcescens	R	S	S	S	S
AR-0099	Serratia marcescens	R	S	I	I	S
AR-0121	Serratia marcescens	R	S	I	S	S
AR-0122	Serratia marcescens	R	S	I	S	S
AR-0123	Serratia marcescens	R	S	I	S	S
AR-0124	Serratia marcescens	R	S	I	S	S
AR-0130	Serratia marcescens	R	S	R	S	S
AR-0131	Serratia marcescens	R	S	R	S	S
AR-0517	Serratia marcescens	R	R	R	R	R
AR-0520	Serratia marcescens	R	R	I	R	R
AR-0521	Serratia marcescens	R	R	R	R	R
AR-0608	Serratia marcescens	R	I	R	R	S
UF-166	Serratia marcescens	R	R	R	S	R
UF-263	Serratia marcescens	R	S	R	S	R
UF-269	Serratia marcescens	R	S	R	S	R

Minimal inhibitory concentration (MIC) ug/mL, pattern of susceptibility resistant (R), intermediate (I), and susceptible (S).
